# The blood–brain barrier after stroke: Structural studies and the role of transcytotic vesicles

**DOI:** 10.1177/0271678X16629976

**Published:** 2016-01-28

**Authors:** Michael J Haley, Catherine B Lawrence

**Affiliations:** Faculty of Life Sciences, University of Manchester, Manchester, UK

**Keywords:** Astrocyte end-feet, blood–brain barrier, caveolae, cerebral ischaemia, obesity

## Abstract

Blood–brain barrier breakdown worsens ischaemic damage, but it is unclear how molecules breach the blood–brain barrier in vivo. Using the obese *ob/ob* mouse as a model of enhanced blood–brain barrier breakdown, we investigated how stroke-induced structural changes to the microvasculature related to blood–brain barrier permeability. *Ob/ob* mice underwent middle cerebral artery occlusion, followed by 4 or 24 h reperfusion. Blood–brain barrier integrity was assessed using IgG and horseradish peroxidase staining, and blood–brain barrier structure by two-dimensional and three-dimensional electron microscopy. At 4 and 24 h post-stroke, *ob/ob* mice had increased ischaemic damage and blood–brain barrier breakdown compared to *ob/–* controls, and vessels from both genotypes showed astrocyte end-foot swelling and increased endothelial vesicles. *Ob/ob* mice had significantly more endothelial vesicles at 4 h in the striatum, where blood–brain barrier breakdown was most severe. Both stroke and genotype had no effect on tight junction structure visualised by electron microscopy, or protein expression in isolated microvessels. Astrocyte swelling severity did not correlate with tissue outcome, being unaffected by genotype or reperfusion times. However, the rare instances of vessel lumen collapse were always associated with severe astrocyte swelling in two-dimensional and three-dimensional electron microscopy. Endothelial vesicles were therefore the best spatial and temporal indicators of blood–brain barrier breakdown after cerebral ischaemia.

## Introduction

The blood–brain barrier (BBB) is a series of specialised structures located in the cerebrovasculature that tightly control the passage of molecules between the blood and brain parenchyma. After cerebral ischaemia the integrity of the BBB is compromised, allowing uncontrolled entry of molecules into the brain parenchyma that worsens damage caused by ischaemia. In patients, a loss of BBB integrity is associated with worse stroke outcome.^1,2^ Ischaemia can affect all components of the BBB, including endothelial cells, astrocytes, pericytes and the extracellular matrix, to negatively affect survival. However, the exact processes occurring in vivo that allow molecules to pass through the BBB into the brain during ischaemia are unclear.

Endothelial cells are the primary barrier to the entry of blood-borne products into the central nervous system (CNS), possessing tight junction proteins that limit paracellular diffusion and tightly regulated transcellular transport. The current view is that ischaemia triggers the active disassembly of tight junctions, thus increasing paracellular diffusion. In vitro studies suggest hypoxia-reperfusion injury affects the subcellular localisation of the proteins zona occludens 1, occludin and claudin-5 and their association with tight junction complexes.^3–5^ However, when studied in vivo in an embolic model of stroke, tight junctions appear intact even in vessels showing BBB breakdown.^6^ Similar findings have been made recently in a transgenic mouse strain whose endothelial tight junctions are labelled with eGFP, allowing the dynamics of tight junction integrity to be monitored longitudinally in vivo*.*^7^ In these mice, tight junctions remain intact within the infarct until at least 24 h post-stroke, despite tracer accumulating in the brain within 6 h. Rather than being due to disruption of tight junctions, the loss of BBB integrity is associated with an increase in endothelial vesicles identified as caveolae.^6–8^ Rats undergoing ischaemic stroke possessed a BBB that was permeable to the tracer Evans blue, but with no change in its permeability to ions, suggestive of a transcellular rather than paracellular opening of the BBB.^9^ Qualitative increases in transcytotic transport across the BBB have been reported in several models of cerebral ischaemia.^10–15^ However, although endothelial vesicles increase after cerebral ischaemia and can contribute to BBB breakdown, their overall impact on BBB integrity is unclear. If endothelial vesicles are important for BBB breakdown, their numbers would be expected to correlate spatially and temporally with BBB breakdown.

Pericytes and astrocytes envelop endothelial cells but do not themselves constitute a barrier to solutes, instead promoting BBB integrity through their interactions with endothelial cells. Pericytes are thought to promote endothelial barrier function by increasing tight junction integrity^16–18^ or limiting the rate of endothelial transcytosis.^19^ Some pericytes also possess alpha-smooth muscle actin capable of contracting after stroke, potentially contributing to the ‘no-reflow’ phenomenon after cerebral ischaemia, in which blood flow does not successfully return to capillaries in the brain after recanalization of larger vessels.^20,21^ Astrocytes may also contribute to ‘no-reflow’ because after stroke astrocytes surrounding blood vessels can rapidly become oedematous, potentially compressing the vessel lumen and limiting reperfusion of blood.^22–24^

In the current study, we investigate how the structure of the microvasculature is affected by experimental stroke, and whether these structural changes relate to transcellular and paracellular BBB permeability. Worse BBB damage after stroke has been previously reported in obese *ob/ob* mice,^25^ and in other obese rodents.^26–29^ Due to the large difference in both severity and volume of BBB breakdown between *ob/ob* and controls, the obese *ob/ob* mouse was therefore used as a model in which to study mechanisms by which BBB permeability is mediated. In this way, the *ob/ob* mouse was used primarily as a model of enhanced BBB damage, rather than as a model of the impact of obesity on stroke outcome. The structure and the permeability of the BBB were compared between genotypes, brain regions and at different reperfusion times after experimental stroke.

## Materials and methods

### Animals

Male obese *ob/ob* (C57BL/6 OlaHsd-Lep^ob^) and control (*ob/–*) mice were supplied by Harlan (UK). The weight at the time of surgery was 53.7 ± 6.8 g for the obese (*ob/ob*) mice, and 32.1 ± 5.6 g for control (*ob/–*) mice (mean ± s.d.). The age range of both genotypes was 16 to 19 weeks. A total of 29 *ob/–* mice were used, of which 2 died during surgery and 1 was excluded due to subarachnoid haemorrhage, and 31 *ob/ob* mice, of which 2 died during surgery and 1 upon recovery, and 2 were excluded due to subarachnoid haemorrhage.

Mice were housed in cages of four or five in standard housing conditions (temperature, 20℃ ± 2℃; humidity, 55% ± 5%; 12-h light/12-h dark cycle), and given free unlimited access to standard rodent chow (RM1, Special Diet Services, UK) and water. All studies were conducted in accordance with the UK Animals (Scientific Procedures) Act 1986 and approved by the Home Office and the local Animal Ethical Review Group, University of Manchester and reported in compliance with the ARRIVE guidelines. Animals were assigned randomly to experimental groups (naïve or middle cerebral artery occlusion (MCAO), and 4 or 24 h reperfusion).

### Middle cerebral artery occlusion

Focal cerebral ischaemia was induced in the left cerebral hemisphere (ipsilateral) by MCAO.^30^ Under isoflurane anaesthesia (2% in 200 ml/min O_2_ and 500 ml/min N_2_O), an intraluminal filament (filament diameter 70 µm, silicon coating diameter 210 µm, coating length 4–5 mm, Doccol, USA) was advanced via the left external carotid artery into the internal carotid artery until it occluded the middle cerebral artery (MCA) at its origin. After 20 min occlusion, the filament was withdrawn to allow reperfusion. Cerebral blood flow (CBF) was monitored throughout using a laser-flow blood perfusion monitor connected to a laser-Doppler flow probe (Moor instruments, UK) attached to the animal’s skull overlying the territory supplied by the MCA. Successful occlusion was confirmed by a reduction of at least 80% in CBF. Body temperature was maintained at 37℃ using a homeothermic heat mat (Harvard Apparatus, UK).

### Tissue processing

Mice were perfused intracardially under isoflurane anaesthesia at 10 ml/min. For preparation of isolated microvessels, mice were perfused with 0.9% saline, their brains removed and snap frozen in isopentane on dry ice. Microvessels were isolated from whole brains as previously described.^31^ For measurement of ischaemic damage and IgG leakage, mice were perfused with 0.9% saline followed by fixative (4% paraformaldehyde (PFA), in 0.1 M phosphate buffer (PB)), after which brains were removed, post-fixed, cryoprotected and frozen in isopentane on dry ice. Sections were then cut at 30 µm intervals on a freezing sledge microtome (Bright Instruments, UK).

For electron microscopy (EM), mice were perfused with 0.9% saline followed by fixative (2% PFA and 2.5% glutaraldehyde in 0.1 M PB), after which brains were removed and post-fixed for 6 h. Slices were cut on a vibrating blade microtome (100 µm thick for transmission electron microscopy (TEM), 50 µm for visualisation of horseradish peroxidase (HRP) by TEM, and 1 mm for serial block-face scanning electron microscopy), selected areas of interest dissected out (Figure 3(g)) and processed for EM as previously described.^32^ Briefly, tissues were fixed for 1 h on ice with 1.5% potassium ferrocyanide and 2% osmium tetroxide (weight/vol) in 0.1 M cacodylate buffer. This was followed by incubations with 1% thiocarbohydrazide for 20 min at room temperature, 2% osmium tetroxide for 30 min at room temperature and 1% uranyl acetate at 4℃ overnight. The next day, samples were stained with freshly prepared Walton’s lead aspartate (0.02 M in lead nitrate and 0.03 M in aspartic acid, adjusted to pH 5.5) for 30 min and embedded in Epon 812 (hard formulation) epoxy resin (Electron Microscopy Science, UK).

### TEM

For TEM, ultrathin sections (70 nm) were cut from resin-embedded samples on an ultramicrotome (Leica), mounted on Formvar-coated grids and viewed on a FEI Tecnai 12 Biotwin Transmission Electron Microscope. For each specimen, images of the first 15 capillaries identified were collected digitally using a Gatan Orius SC1000 camera.

### Serial block-face scanning electron microscopy

Resin-embedded samples were attached to cryo specimen pins, trimmed to form a trapezoid face approximately 500 × 500 × 150 µm and the block-face polished on an ultramicrotome. Samples were mounted on a Gatan 3view within an FEI Quanta 250 FEG, a system which allows back-scattered electron images of the block surface to be collected, as the sample is microtomed in situ.^33^ This allows serial section TEM-like images to be collected in an automated fashion. The imaging settings were accelerating voltage: 3.8 kV, spot size: 3.5, final lens aperture: 30 µm, chamber pressure: 66 Pa, quadrant magnification: 3500 (giving a horizontal field width of approximately 40 µm), image dimensions: 4096 by 4096, pixel dwell time: 10 µs and cut thickness: 100 nm. Raw data were converted to an MRC file format stack using IMOD software and processed as previously described.^34^ Data were visualised, and pericytes, endothelial cells and astrocyte end-feet swelling manually segmented using Amira (FEI Visualization Sciences, France). Areas of astrocyte swelling were defined as areas of increased end-feet size, with a sparse cytoplasm possessing low electron density (appearing white on micrographs). Non-swollen (healthy) astrocyte end-feet were not segmented.

### EM quantification

Micrographs of capillaries were collected from four regions; the contralateral (non-ischaemic) and ipsilateral (ischaemic) striatum, and contralateral and ipsilateral cortex (*n* = 3–5 mice per group, 15 vessels per region in each mouse). On each micrograph, the following measurements were made using ImageJ software (NIH, USA): lumen average and minimum diameter, lumen circularity, pericyte coverage, tight junction tortuosity, basal lamina thickness, astrocyte swelling score, number of endothelial vesicles and endothelial cell area. For each parameter and region, measurements from 15 vessels were averaged to give a single biological repeat (*n* number). Lumen circularity was calculated using the ImageJ circularity function $(\mathrm{circularity}=4\pi (\frac{\mathrm{area}}{\mathrm{perimeter}}{)}^{2})$, with a value of 1 indicating a perfect circle. Pericyte coverage was calculated as the percentage of the lumen circumference covered by either a pericyte cell body or process.^35^ Tight junction tortuosity was used as a measure of tight junction complexity and was calculated as previously described; the measured length of the tight junction from where it starts at the luminal side and ends at the basal lamina was divided by the diagonal of the rectangle that contains the complete tight junction.^36^ Basal lamina thickness was measured in three equidistant locations and an average taken. Astrocyte swelling was scored on a scale of zero to five based on the amount of basal lamina covered by the adjoining swollen astrocyte end-feet, and how severe the swelling was in terms of area. Zero indicated no swelling, one to four increasing severities of swelling, and five severe swelling that was visibly compressing the vessel lumen. Endothelial vesicles were counted in each capillary micrograph and data expressed as number of vesicles per µm of vessel wall. Endothelial cell area was measured, and divided by the lumen diameter to account for variation in vessel size.

### Assessment of ischaemic damage and haemorrhagic transformation

Ischaemic damage was measured on coronal brains sections stained with haematoxylin and eosin (H&E), with infarct volume calculated by measuring the areas of neuronal loss at eight defined coronal levels as previously described.^37^ On each section, damage was measured over the entire ipsilateral hemisphere, and also specifically in the cortex, striatum and ‘other’ areas (hippocampus and thalamus). To quantify haemorrhagic transformation, the area of red blood cells was measured in the same sections used to calculate ischaemic damage.

### BBB permeability to IgG

To assess BBB permeability, IgG in the brain was visualised by peroxidase-based immunohistochemistry. Endogenous peroxidase activity and non-specific staining were blocked, and sections incubated in biotinylated anti-mouse IgG secondary antibody (1:500, Vector Laboratories) overnight at 4℃. Sections were then incubated with avidin–biotin–peroxidase complex, and colour-developed using a diaminobenzidine (DAB) solution (0.05% DAB in 0.005% H_2_O_2_). To ensure comparability of DAB staining, all reactions were performed at the same time, with the same batch of DAB, with equal thickness sections exposed to DAB for the same amount of time. IgG staining intensity (using ImageJ) was averaged over eight coronal sections (at the same levels as assessed for ischaemic damage), with intensity increases in the ipsilateral hemisphere expressed as percentage increase compared to equivalent areas in the contralateral hemisphere. All sections were imaged at the same time with the same settings, and with no adjustment to brightness or contrast. IgG staining was measured in the entire cortex, striatum and ‘other’ (hippocampus and thalamus) areas.

### Horseradish peroxidase injection

Mice were injected via the tail vein with 10 mg per 20 g of HRP type II (44 kDa, Sigma, UK) and at 2 h post-injection, prepared for EM as described above. After 50 µm sections were cut as described, HRP was visualised by incubating sections with DAB solution (0.05% DAB in 0.05 M Tris-HCl, pH 8.0) for 20 min followed by replacement with DAB solution containing 0.02% H_2_O_2_ for 20 min. For visualisation of HRP by EM, samples were then dissected from regions of interest (Figure 3(g)) processed for EM and viewed by TEM (described above). For light microscopy, sections were mounted and coverslipped on slides using DPX.

### Immunoblotting

Antibodies for immunoblotting were as follows: anti-claudin-5 (1:1000, Abcam), anti-occludin (1:500, Life Technologies) and anti-β-actin (1:5000, Sigma). Microvessel samples were homogenised in buffer (50 mM Tris-HCl, 150 mM NaCl, 5 mM CaCl_2_, 0.02% NaN_3_, 1% Triton X-100), separated by SDS page and proteins transferred to a polyvinylidene fluoride membrane. After being blocked (5% milk and 0.1% TWEEN-20 in PB-Saline (PBS-T)), membranes were incubated at 4℃ overnight in primary antibodies diluted in 1% BSA in PBS-T. Membranes were then incubated with HRP-conjugated secondary antibodies (1:500, Wako Chemicals) in 5% milk in PBS-T and blots developed using an Enhanced Chemiluminescent Detection Kit (GE Healthcare). Digital images of protein bands were acquired (ImageQuant 350, GE Healthcare, UK) and semi-quantitative analysis of protein content performed by densitometry using ImageQuant TL software (GE Healthcare, UK), with β-actin used as a loading control.

### Data and statistical analyses

All data are presented as mean ± standard error of the mean (S.E.M). Sample sizes were determined by power calculation (α = 0.05, β = 0.2) of our previous data. All ex vivo quantification was done blinded to genotype, time point, treatment (naïve vs MCAO) and brain region. All statistical tests were performed using SigmaStat (Systat, USA) or GraphPad Prism (GraphPad, USA) software, with *P* < 0.05 considered as significant. For evaluation of the response to stroke in obese *ob/ob* mice, ischaemic damage for each brain region was analysed by Mann–Whitney tests and IgG staining by Student’s *t*-tests, both with Sidak-Bonferroni correction for multiple comparisons. Area of blood cells was analysed by Mann–Whitney U test. Other comparisons were made by two-way analysis of variance with Sidak-Bonferroni post hoc test.

## Results

### Increased and earlier ischaemic damage and haemorrhagic transformation in obese ob/ob mice

**Figure 1.**
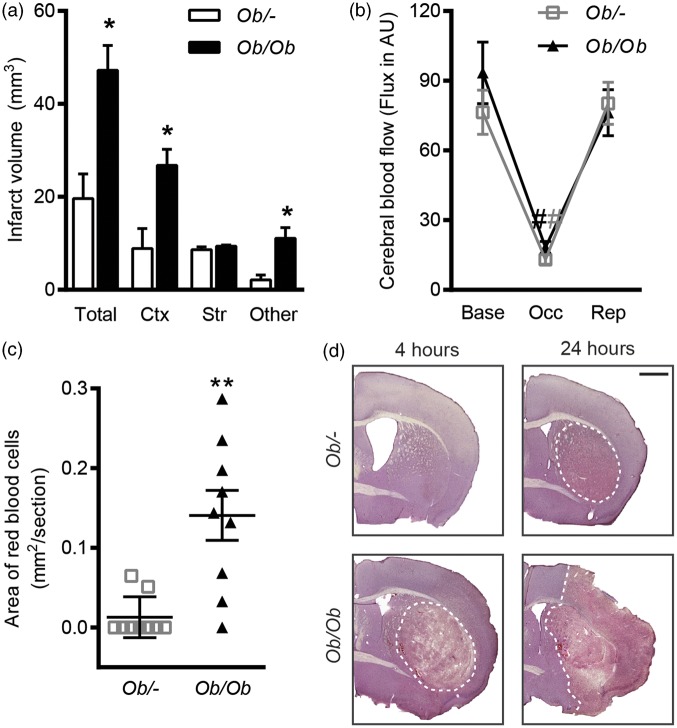
Increased and earlier ischaemic damage after middle cerebral artery occlusion (MCAO) in obese *ob/ob* mice. (a) Total infarct volume (ischaemic damage) at 24 h was greater in obese *ob/ob* mice due to increased damage in cortical (Ctx) and ‘other’ (hippocampus and thalamus) areas but was not different in the striatum (Str) compared to control *ob/–* mice. (b) When measured by laser-Doppler flowmetry, cerebral blood flow was not different to controls in obese *ob/ob* mice at baseline (Base), during occlusion (Occ) or at reperfusion (Rep). (c) Haemorrhagic transformation was worse 24 h after MCAO in obese *ob/ob* mice as indicated by an increase in the area of red blood cells. (d) Representative haematoxylin and eosin-stained sections from obese *ob/ob* and control *ob/–* mice at 4 and 24 h post-MCAO indicating ischaemic damage and haemorrhagic transformation are detected as early as 4 h in obese mice. Dotted line indicates the lesion area. Scale bar = 1 mm. Data are mean ± S.E.M., *n* = 9 per group, *P < 0.05 and **P < 0.01 versus control *ob/–*, #*P* < 0.05 versus baseline and reperfusion.

At 24 h reperfusion after 20 min, MCAO obese *ob/ob* mice had a 2.4-fold increase in total ischaemic damage and an 11-fold increase in area of haemorrhagic transformation when compared to *ob/–* controls (Figure 1(a) and (c)). As striatal damage was not significantly different between genotypes, this difference in total ischaemic damage was due to an increase in cortical, and thalamic and hippocampal (‘other’) damage in obese *ob/ob* mice. Striatal damage and haemorrhagic transformation were already evident at 4 h post-stroke in obese *ob/ob* mice, but was absent in controls (Figure 1(d)). Measurement of CBF in the MCA territory by laser-Doppler flowmetry found no differences between genotypes at baseline or in response to MCA occlusion (Figure 1(b)).

### Enhanced and earlier BBB damage in obese ob/ob mice

**Figure 2.**
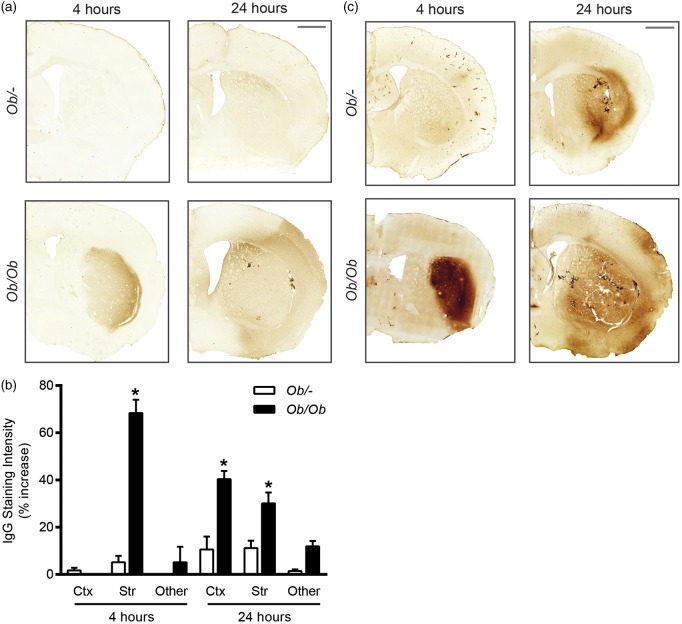
Increases in blood–brain barrier (BBB) permeability to IgG and horseradish peroxidase (HRP) occur quicker and were more severe after middle cerebral artery occlusion (MCAO) in obese *ob/ob* mice. (a) Representative IgG-stained sections from obese *ob/ob* and control *ob*/– mice at 4 and 24 h post-MCAO. (b) At 4 h post-MCAO, obese *ob/ob* mice had a significantly greater loss of BBB integrity to IgG in the striatum (Str). At 24 h post-MCAO, BBB integrity to IgG was also significantly worse in the striatum and cortex (Ctx) of obese *ob/ob* mice. No significant difference was seen in the hippocampus and thalamus (‘other’). Values are expressed as percentage increase in the ipsilateral hemisphere compared to the equivalent areas in the contralateral hemisphere. (c) The permeability of the BBB to HRP after stroke was increased in obese *ob/ob* mice, being greatest at 4 h post-MCAO in the striatum. At 24 h post-stroke, the area of permeability to HRP is similar to the area of ischaemic damage in both genotypes, with HRP being mostly detectable in the striatum of control mice, but also in the cortex of obese *ob/ob* mice. HRP was injected via the tail vein 2 h prior to culling. Scale bars = 1 mm. For IgG, data are mean ± S.E.M., *n* = 3 at 4 h and *n* = 9 at 24 h/genotype, *P < 0.05 versus same brain region in control *ob/–* mice. For HRP; *n* = 3/time point and genotype.

Ischaemic disruption of the BBB was measured initially by infiltration of endogenous IgG into the brain parenchyma (Figure 2(a) and (b)). At both 4 and 24 h post-MCAO, obese *ob/ob* mice had a greater degree of BBB disruption as a significant increase (*P* < 0.05) in IgG staining intensity was observed in the ipsilateral hemisphere compared to control mice. At 4 h post-MCAO, this increase in IgG staining was confined to the striatum, whereas at 24 h IgG staining was greater in the cortex and striatum of obese *ob/ob* mice.

**Figure 3.**
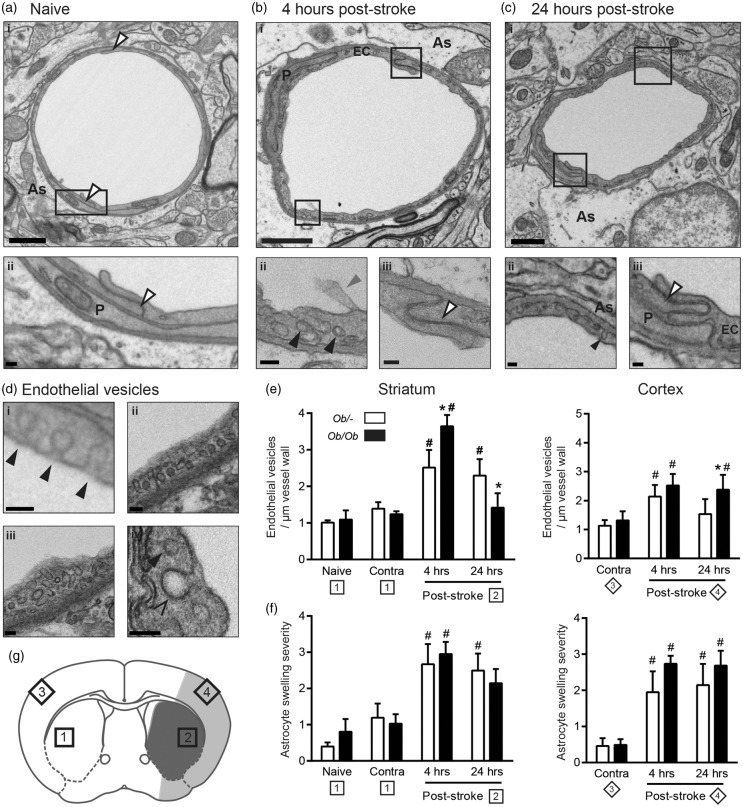
Stroke resulted in swelling of astrocyte end-feet, and an increase in endothelial vesicles that was greater in obese mice. Electron micrographs are representative of vessels from the striatum of naive mice (a), and at 4 (b) and 24 (c) hours post-middle cerebral artery occlusion (MCAO). Qualitatively vessels from the cortex and striatum appeared identical, as did vessels from *ob/*– and obese *ob/ob* mice. At both 4 (b) and 24 (c) hours post-MCAO, astrocytes were often oedematous and numerous vesicles were present in endothelial cell cytoplasm (bii, cii). The quantified vesicles (di, ii, iii) were readily distinguishable from clathrin-coated pits (div) or vesicles by size and lack of an electron dense coat. They could also be seen forming on the luminal surface (di), and fusing with the abluminal surface (cii) of endothelial cells. At both 4 and 24 h post-stroke, tight junctions do not appear to be disrupted (biii, ciii). Endothelial vesicle numbers were increased in the ipsilateral relative to the contralateral (Contra) hemisphere, and were greater in the striatum and cortex of obese *ob/ob* mice at 4 (for striatum) and 24 (for cortex) h post-stroke (e). Astrocyte swelling severity was significantly worse at 4 and 24 h after MCAO in the ipsilateral compared to contralateral cortex and striatum in both control *ob/–* and obese *ob/ob* mice (f). Data are mean ± S.E.M., *n* = 3–5 per group, *P < 0.05 versus control *ob/–* at same time point, #*P* < 0.05 versus contralateral of same genotype. Sampling sites for contralateral (1) and ipsilateral (2) striatum, and contralateral (3) and ipsilateral cortex (4) are indicated. Shaded areas indicate areas confirmed as infarcted by H&E at 24 h in control *ob*/– (dark grey only; striatum) and *ob/ob* (dark and light grey; striatum and cortex) mice (g). Labelled structures include pericytes (P), endothelial cells (EC), astrocyte end-feet (As), tight junctions (white arrows), vesicles (black arrows), clathrin-coated pit (open arrow head) and microvilli (grey arrow). Scale bars are 1 µm in large images (ai, bi and ci), 100 nm in their insets, and 100 nm in D.

Since the exact time of entry of IgG detected in the brain at 24 h cannot be confirmed, the permeability of the BBB at specific times post-MCAO was assessed by intravenous HRP injection. HRP was administered at 4 or 24 h post-stroke and its presence in the brain parenchyma was detected by the DAB reaction (Figure 2(c)). At 24 h post-stroke, the area of DAB staining due to the presence of HRP appeared greater in obese *ob/ob* mice, extending into the cortex, whereas in control *ob/–* mice staining was predominantly confined to the striatum. Staining intensity at 24 h was similar between genotypes. At 4 h post-stroke, there was a marked difference in HRP staining between genotypes, being barely detectable in control *ob/*– mice, but intense and well defined in the striatum of obese *ob/ob* mice. The pattern of HRP staining at both 4 and 24 h correlated with the areas of ischaemic damage detected by H&E staining (Figure 1(d)). In some instances HRP staining was associated with the vasculature, but HRP that penetrated into the parenchyma appeared diffuse and not specific to any particular cellular structure.

### Stroke resulted in an increase in endothelial vesicles and astrocytic swelling

Stroke had pronounced effects on the structure of the BBB at both 4 and 24 h post-MCAO, in both mouse genotypes (Figure 3(a) to (c)). Qualitative assessment showed a large increase in the number of endothelial vesicles and swelling of astrocyte end-feet in the ischaemic area that was not seen in naïve mice. Varying degrees of damage to the tissue surrounding vessels were also evident post-stroke, often featuring necrotic cell death, vacuoles and cellular debris. Vessels from the cortex and striatum appeared identical, as did vessels from *ob/*– and obese *ob/ob* mice. No obvious effects of stroke or genotype were observed on the structure of tight junctions even in severely damaged endothelial cells. In animals used for EM, H&E staining confirmed that at 4 h striatal damage was evident in obese *ob/ob* but not *ob/–* control mice. At 24 h *ob/–* control mice had striatal damage only, whereas obese *ob/ob* mice had damage in the striatum and the cortex.

In response to MCAO, endothelial vesicles were readily identifiable within the cytoplasm of endothelial cells, as were flask-shaped invaginations on both the luminal and abluminal surfaces of endothelial cells (Figure 3, bii, cii, di). The vesicles counted were readily distinguishable from clathrin-coated vesicles (Figure 3 d) due to their lack of an electron dense coat and smaller size (typically 50 nm diameter), consistent with vesicles identified previously as caveolae.^7^ Furthermore, in areas of more severe tissue damage, endothelial cells often showed cytoplasmic and nuclear swelling, cytoplasmic vacuoles and microvilli forming on the lumen wall. However, neither nuclear changes associated with cell death (apoptotic or necrotic) nor overt cytoplasmic disintegration were observed in endothelial cells. Stroke resulted in a significant upregulation of endothelial vesicles in both genotypes; however, significantly (*P* < 0.05) more vesicles were found in obese *ob/ob* mice in the striatum at 4 h and cortex at 24 h (Figure 3(e)). These increases in endothelial vesicles correlated with increases in BBB permeability to IgG and HRP in both their timing post-stroke, and location in the brain.

**Figure 4.**
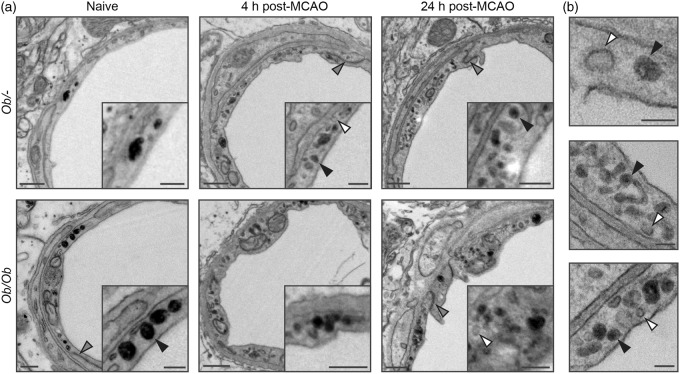
Horseradish peroxidase- (HRP) filled vesicles were found in the cytoplasm of endothelial cells (black arrows) but not in the vicinity of tight junctions. (a) The number of HRP-positive vesicles was low in naïve animals, but appeared to increase at both 4 and 24 h post-stroke in both control *ob/–* and obese *ob/ob* mice. (b) Further examples of endothelial vesicles post-stroke. Not all vesicles were HRP-filled (white arrows). No obvious deposition of HRP in the basal lamina was found in the vicinity of tight junctions, which appeared intact (grey arrows). Scale bars 500 nm in (a), 250 nm in insets of (a), 100 nm in (b), *n* = 3 per group.

To determine whether endothelial vesicles could take up solutes from the blood, animals were injected intravenously with HRP. Endothelial vesicles containing HRP were found in the cytoplasm of endothelial cells in both naïve control *ob/–* and obese *ob/ob* mice, being distinguishable from HRP-negative vesicles due to their dark staining (Figure 4). At 4 and 24 h post-stroke, the number of HRP-positive endothelial vesicles appeared to be increased, paralleling earlier results of increased vesicles after stroke. The proportion of total endothelial vesicles filled with HRP appeared similar between genotypes but was not quantified. No deposits of HRP were visible in the vicinity of tight junctions in any vessels analysed. HRP that penetrated the BBB did not appear to be concentrated in any particular structure in the parenchyma, and so it was not possible to distinguish confidently HRP in the parenchyma from other electron dense structures (when assessed by EM).

Astrocyte end-feet were often swollen after stroke, visible by EM as areas of diffuse cytoplasm making immediate contact with the basal lamina (Figure 3(a) to (c)). In some cases, this swelling was enough to completely compress the vessel lumen, but this was uncommon. Typically, astrocyte oedema covered at least half the basal lamina and extended between 0.5 and 1 µm into the parenchyma. Whether less severe oedema was also capable of compressing the lumen is difficult to assess in two-dimensional (2D), since the vessels diameter and shape prior to the appearance of swelling are unknown. When the severity of swelling was graded based on the area surrounding each vessel, stroke resulted in an increase in swelling severity in both genotypes in the cortex and striatum at both 4 and 24 h post-MCAO (Figure 3(f)), but no difference in the extent of swelling between control *ob/–* and obese *ob/ob* mice was observed.

When endothelial area was expressed as an absolute value, no significant differences were found between time points or genotypes (Table 1). However, when variability in lumen diameter was accounted for endothelial area was increased at 24 h post-stroke in the cortex of *ob/ob* mice relative to contralateral hemisphere.

**Table 1.** Quantification of vessel characteristics from electron micrographs.

		Striatum				Cortex		
		Naïve	Contra	4 h	24 h	Contra	4 h	24 h
Basal lamina thickness (nm)	*Ob/–*	71.7 ± 2.1	66.9 ± 3.7	79.2 ± 8.1	70.2 ± 6.0	63.4 ± 5.6	85.6 ± 3.3	65.9 ± 7.6
	*Ob/Ob*	70.4 ± 2.8	64.2 ± 1.4	80.5 ± 6.0	71.9 ± 6.0	65.5 ± 3.3	75.9 ± 7.3	65.4 ± 8.1
Pericyte coverage (%)	*Ob/–*	31.0 ± 2.4	33.2 ± 1.7	31.1 ± 4.7	27.9 ± 8.3	24.6 ± 1.9	32.2 ± 2.7 #	32.4 ± 1.2
	*Ob/Ob*	30.7 ± 2.8	32.2 ± 2.7	30.6 ± 13.6	35.4 ± 4.0	29.4 ± 2.3	26.8 ± 3.5 *	33.5 ± 1.7
Tight junction tortuosity	*Ob/–*	1.33 ± 0.13	1.34 ± 0.09	1.51 ± 0.36	1.73 ± 0.35	1.31 ± 0.05	1.29 ± 0.05	1.47 ± 0.13
	*Ob/Ob*	1.40 ± 0.19	1.31 ± 0.16	1.38 ± 0.40	1.26 ± 0.18	1.40 ± 0.12	1.34 ± 0.10	1.24 ± 0.04
Average lumen diameter (µm)	*Ob/−*	5.0 ± 0.1	4.4 ± 0.5	4.8 ± 0.5	4.6 ± 0.7	4.8 ± 0.3	4.0 ± 0.9	4.4 ± 0.8
	*Ob/Ob*	5.8 ± 0.1	5.1 ± 0.2	5.0 ± 0.6	5.2 ± 0.1	4.9 ± 0.1	4.7 ± 0.3	5.2 ± 0.2
Minimum lumen diameter (µm)	*Ob/–*	4.3 ± 0.1	4.0 ± 0.3	3.9 ± 0.3	4.3 ± 0.5	4.1 ± 0.3	3.7 ± 0.4	4.1 ± 0.5
	*Ob/Ob*	4.1 ± 0.2	4.3 ± 0.2	3.7 ± 0.3	4.5 ± 0.2	4.3 ± 0.1	4.1 ± 0.1	4.5 ± 0.2
Lumen circularity	*Ob/–*	0.80 ± 0.03	0.72 ± 0.05	0.72 ± 0.06	0.65 ± 0.05	0.74 ± 0.04	0.64 ± 0.08	0.66 ± 0.05
	*Ob/Ob*	0.75 ± 0.05	0.72 ± 0.04	0.69 ± 0.05	0.76 ± 0.04	0.77 ± 0.02	0.77 ± 0.01	0.69 ± 0.03
Endothelial cell area (µm^2^)	*Ob/–*	25.4 ± 0.9	25.2 ± 1.9	23.2 ± 2.4	28.2 ± 4.1	24.5 ± 2.4	26.2 ± 2.0	24.6 ± 4.0
	*Ob/Ob*	22.3 ± 0.7	25.2 ± 1.5	21.5 ± 2.8	29.2 ± 1.8	23.8 ± 1.3	23.4 ± 1.9	30.4 ± 2.0
Endothelial area/lumen diameter (µm^2^/µm)	*Ob/–*	5.07 ± 0.11	5.23 ± 0.12	4.66 ± 0.20	5.53 ± 0.25	4.99 ± 0.20	5.01 ± 0.16	4.91 ± 0.30
	*Ob/Ob*	4.71 ± 0.12	5.05 ± 0.10	4.78 ± 0.22	5.36 ± 0.14	4.80 ± 0.14	4.81 ± 0.21	5.65 ± 0.13 #

Note: Samples were taken from naïve animals, the contralateral hemisphere (Contra), or at 4 (4 h) or 24 (24 h) hours post-MCAO. Data are mean ± S.E.M., *n* = 3–5 per group). *P < 0.05 versus control *ob/−* at same time point and area, #*P* < 0.05 versus contralateral of same genotype and area.

With the exception of an increase in pericyte coverage at 4 h post-stroke in *ob/–* controls, no other differences were found in any other measured parameters (lumen average and minimum diameter, lumen circularity, pericyte coverage, tight junction tortuosity and basal lamina thickness) either in naïve animals or in response to stroke at 4 or 24 h (Table 1).

### Three-dimensionalvisualisation of endothelial cells, astrocyte swelling and pericytes post-stroke

**Figure 5.**
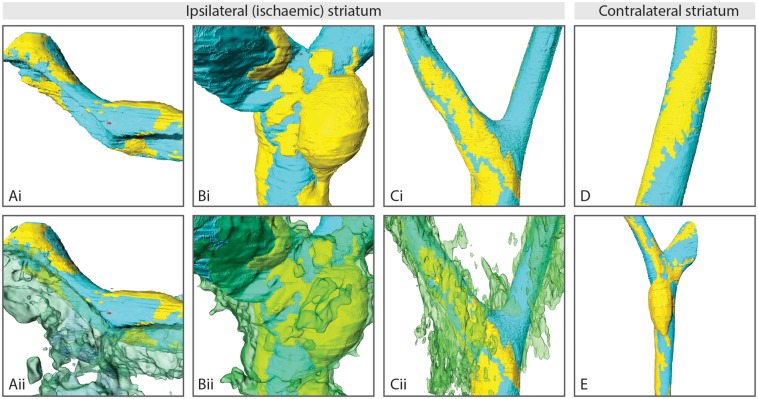
Visualisation of cerebral capillaries post-stroke, showing endothelial cells, pericytes and astrocyte end-foot swelling. Endothelial cells (teal), astrocyte swelling (transparent green) and pericytes (yellow) were manually segmented on serially sectioned electron micrographs of striatal capillaries, giving a Z-stacked volume which was rendered in 3D. Astrocyte end-feet swelling in the ischaemic (ipsilateral) striatum was associated with almost complete lumen collapse (a, 4 h, *ob/ob*) and partial compression (b, 4 h, *ob/–*), though some vessels showed a patent lumen despite extensive astrocyte swelling (c, 24 h, *ob/ob*). Capillaries from the contralateral striatum had healthy astrocytes with no swelling and thus no transparent green channel is displayed (d, 24 h, *ob/–* and e, 24 h, *ob/ob*). See supplementary material for videos.

Serial block-face SEM allowed the effects of stroke on the structure of the BBB to be rendered in three-dimensional (3D) and assessed qualitatively (Figure 5). In agreement with 2D EM data, all ipsilateral areas studied (4 and 24 h post-stroke, in obese *ob/ob* and *ob/–* control mice, in the striatum and cortex) showed varying severities of astrocyte swelling. Areas of almost complete collapse (Figure 5(a)) or partial compression of the lumen (Figure 5(b)) were associated with astrocyte swelling. Other vessels showed little lumen compression despite extensive astrocyte swelling nearby (Figure 5(c)). However, in all instances where the lumen was obviously compressed or collapsed astrocyte swelling was present. In vessels from contralateral (Figure 5(d) and (e)) and naïve areas, astrocyte end-feet were not swollen, and the lumen had a relatively uniform diameter across the measured length.

Pericytes appeared similar between contralateral (Figure 5(d) and (e)) and ipsilateral areas (Figure 5(a) to (c)), possessing processes that ran longitudinally down vessels, rather than circumferentially. However, after stroke the shape of pericyte cell bodies appeared to become less elongated, and more amoeboid (Figure 5(b)). Despite this change in nuclear shape, pericytes remained closely associated with endothelial cells. Furthermore, there was no evidence of nuclear changes that would suggest pericytes were dying of either apoptosis or necrosis in any micrographs analysed. No obvious decrease in lumen diameter was found in regions underlying pericyte cell bodies, and pericytes appeared similar between control and obese mice.

### Tight junction protein expression in isolated microvessels was unaffected by stroke

**Figure 6.**
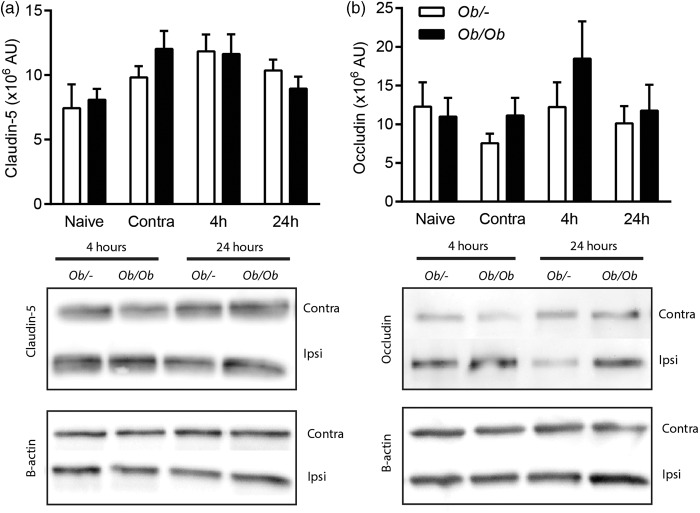
Stroke did not affect the expression of tight junction protein in brain microvessels. Western blot assays for claudin-5 (a) and occludin (b) were performed on microvessel homogenates purified from brains of obese *ob/ob* and control *ob/–* mice. Microvessels were isolated from both hemispheres of naïve animals, and the contralateral (contra) and ipsilateral (ipsi) hemispheres at 4 and 24 h after MCAO. Data are mean ± S.E.M., *n* = 6 per group.

The expression of the tight junction proteins claudin-5 and occludin were measured by Western blot in isolated microvessels from brains of control and obese *ob/ob* mice at 4 or 24 h post-stroke (Figure 6). No significant effect of stroke was found on the expression of either claudin-5 or occludin in the ipsilateral hemisphere when compared to expression in contralateral hemisphere. Neither was there any significant effect of genotype.

## Discussion

Cerebral ischaemia is known to disrupt BBB integrity, yet the mechanisms that mediate this increase in vascular permeability in vivo are unclear. The most well-studied hypothesis is that tight junctions undergo disassembly, resulting in increased paracellular permeability.^38^ Potential transcellular routes across the BBB after ischaemia have been less well explored. The *ob/ob* mouse has been used previously to model the effects of obesity on stroke outcome as it becomes obese due to its lack of leptin.^25,39^ However, rather than as a model of obesity, the *ob/ob* mouse was used here as a model of known enhanced ischaemic BBB damage in which to study what changes in vascular structure correlated spatially and temporally with BBB breakdown.

As previously reported,^25^ obese *ob/ob* mice had exacerbated BBB damage after MCAO, as shown by increased IgG extravasation and an increased incidence of haemorrhagic transformation. We demonstrate here for the first time that this exacerbation occurs early, with HRP extravasation in the brain appearing greatest at 4 h reperfusion in obese *ob/ob* mice. At this time, there was a marked difference in striatal BBB permeability to both HRP and IgG between obese *ob/ob* and control *ob/–* mice. Likely due to the difference in amount of ischaemic damage between genotypes, there was also a spatial difference in BBB permeability, as BBB permeability was relatively low in both genotypes at 4 h in the cortex, and only increased in obese *ob/ob* mice at 24 h. Thus, we hypothesised that mechanisms potentially involved in BBB disruption would correlate with this difference in barrier permeability between genotypes, brain areas and reperfusion times.

Recent studies have demonstrated that cerebral ischaemia increases the number of caveolae and similar endothelial vesicles in the cytoplasm of brain vascular endothelial cells, and these vesicles are capable of transporting molecules across the BBB into the brain.^6,7^ However, it has not been shown previously whether the number of endothelial vesicles or caveolae correlates with the severity of BBB damage which would suggest they had a functional role in mediating BBB permeability. In the present study, the number of endothelial vesicles was increased post-MCAO in both control *ob*/– and obese *ob/ob* mice. Both the greatest numbers of endothelial vesicles and greatest permeability to HRP were found in the striatum of obese *ob/ob* mice at 4 h reperfusion. Control mice had comparatively less BBB breakdown at this time in the striatum and had significantly less endothelial vesicles compared to obese *ob/ob* mice. As there is no specific receptor for HRP on endothelial cells, the uptake of HRP into endothelial vesicles is fluid phase and therefore non-specific.^40^ Therefore, visualisation of HRP within endothelial vesicles suggests other solutes of similar size may also undergo transport by this route. Together, these data suggest that an upregulation of endothelial vesicles may contribute to the early increase in BBB permeability after cerebral ischaemia, and that the number of endothelial vesicles may relate to BBB breakdown severity.

However, the presence or lack of an upregulation of endothelial vesicles did not always predict BBB permeability. At 24 h reperfusion in the striatum of obese *ob/ob* mice, there was no significant increase in endothelial vesicles. This may be because the striatum becomes the core of the infarct where ischaemic damage is most severe, potentially leading to endothelial being unable to maintain vesicular transport. In cases of severe endothelial damage, a loss of cytoplasmic integrity may also contribute to BBB disruption.^6,41,42^ In the present study, endothelial cells in ischaemic areas often appeared qualitatively unhealthy; however, there was no evidence of endothelial cells undergoing necrosis or apoptosis. Furthermore, HRP was only seen penetrating the BBB in vesicles, rather than being freely penetrating the endothelial cytoplasm, and endothelial oedema was not consistently associated with ischaemia. Therefore, an overt loss of endothelial integrity did not appear to mediate the observed increase in BBB permeability in the present study. This disparity is likely due to the less severe ischaemic challenge used here than in previous studies of severe endothelial damage.^6,42^

Conversely, an increase in endothelial vesicles in more healthy penumbral regions does not necessarily result in BBB disruption. For example, endothelial vesicles were increased in control animals at 4 h in the cortex and striatum despite there being very little IgG or HRP leakage. Therefore, although the appearance of capillary endothelial vesicles often correlates with BBB permeability, and vesicles are capable of mediating transcellular transport, it is difficult to extrapolate these findings to gross changes in BBB permeability over large brain regions. This would require assessment of endothelial vesicles over a wider area and in vessels of different sizes.

Based on their appearance by EM, we hypothesise that the endothelial vesicles quantified here were primarily caveolae. Knowland et al.^7^reported that vesicle populations increasing in the mouse vasculature after stroke were predominantly caveolin-1 positive. However, although caveolin-1 knock out mice had significantly reduced endocytosis and BBB permeability, neither endocytosis nor increases in BBB permeability were completely abolished in these animals, suggesting caveolin-1 - and clathrin-independent endocytosis also contributes to transcellular permeability. In the present study, we cannot exclude the possibility that vesicles formed from clathrin- and caveolae-independent endocytotic pathways were also quantified. Such pathways have not been well characterised in the brain, but a potential example may be ICAM-1 mediated endocytosis, which is both clathrin- and caveolae-independent.^43,44^ However, ICAM-1 endocytosis is receptor mediated, and clathrin- and caveolae-independent trancytotic pathways in general have not been well studied for their role in fluid phase transcytosis at the BBB. Although clathrin- and caveolae-independent trancytotic pathways have been identified in the periphery, their contribution to overall transendothelial transport is suspected to be low.^45^

The qualitative assessment of tight junctions viewed by EM has previously been cited as evidence for the involvement of tight junctions in BBB permeability in vivo after stroke.^46^ These studies usually show a gap or widening between the membranes of the endothelial cells at the tight junction complex, though this method has been criticised for its subjectivity.^6^ In the present study, no convincing evidence of tight junction opening was found in response to stroke. Furthermore, no leakage of HRP was found in the vicinity of tight junctions in any micrographs, even in obese mice that at 4 h after stroke had severe increases in BBB permeability. Tight junction tortuosity was calculated as a quantitative measure of tight junction complexity that has been shown to correlate with BBB permeability in an in vivo model of BBB disruption.^36^ However, no effect of stroke or obesity was found on either tight junction tortuosity or tight junction protein expression in isolated microvessels. Despite large differences in BBB permeability between control and obese mice in response to stroke (especially at 4 h), none of the observed or measured aspects of tight junctions correlated with BBB integrity. This is in agreement with recent in vivo studies that have found intact tight junctions even in areas of BBB permeability after cerebral ischaemia.^6,7^

Swelling of astrocyte end-feet is thought to contribute to the ‘no-reflow’ phenomenon after cerebral ischaemia, in which blood flow does not successfully return to capillaries in the brain after recanalization of larger vessels.^20,22–24^ In the present study, astrocyte swelling was present at 4 and 24 h post-stroke, which is in agreement with previous studies demonstrating astrocyte swelling appears within hours of ischaemia and often persists beyond 24 h.^11,12,22,47^ The extent of astrocyte swelling has been correlated with the severity of ischaemic damage,^11,41^ and the severity of BBB breakdown.^48^ However, in the present study, astrocyte swelling severity did not differ between control and obese mice despite them having different spatial and temporal development of ischaemic damage. For example, cortical astrocyte swelling was evident in control *ob/*– mice, despite them having no cortical cell death when visualised by H&E staining at 4 or 24 h. This lack of correlation of astrocyte swelling with tissue outcome may be because although astrocyte swelling was often extensive around vessels post-stroke, it rarely caused complete collapse of the lumen in capillaries visualised by 2D or 3D EM. Indeed, stroke did not cause a significant reduction in average or minimum lumen diameter, or in lumen circularity. However, these measures may miss smaller changes in lumen shape which could still be capable of increasing the resistance to passing haematocytes. Detecting such changes in individual vessels is difficult, as we do not know the vessels pre-stroke dimensions. Furthermore, it was clear qualitatively that astrocyte swelling was capable of causing compression when vessels were seen in 3D. Therefore, our data suggest astrocyte swelling may contribute to ‘no-reflow’ by promoting capillary lumen collapse, but even severe astrocyte swelling does not guarantee capillary compromise or tissue infarction. Indeed, mathematic modelling of capillary lumen collapse suggests collapse would also require high reperfusion pressure, blood osmotic pressure and cerebral capillary permeability, and a low value of capillary stiffness.^49^

Pericytes possess a network of fine processes that envelop their neighbouring endothelial cells. It has been hypothesised that contractile fibres in these processes may irreversibly constrict once pericytes die in rigor, hindering capillary reperfusion.^21^ However, in the present study, there was no obvious evidence of lumen compression by circumferential pericyte processes, though processes were often close to areas of compression by oedema due to their abundancy. Furthermore, we observed no nuclear changes in pericytes that would indicate cell death. The lack of any obvious pericyte death and constriction in the present study is likely because the ischaemic insult was less severe than that used in previous work.^21^ Pericyte cell bodies have also been shown to take on an amoeboid shape within 24 h of stroke before migrating away and adopting a microglia-like phenotype.^50^ In the present study, 3D EM suggested that the pericyte cell body adopted an amoeboid morphology as early as 4 h post-stroke, but remained closely associated with vessels at 24 h. Therefore, whether pericyte contribute to ‘no-reflow’ or migrate away from vessels and differentiate may depend on the severity of cerebral ischaemia, and the time post-reperfusion.

In conclusion, an increase in the number of endothelial vesicles was the best indicator of BBB breakdown, correlating spatially and temporally with BBB breakdown severity. These data suggest that transcellular pathways have been underappreciated in their role in mediating BBB breakdown after cerebral ischaemia. The present and previous studies have highlighted the role of endothelial vesicles and caveolae in transcellular transport after cerebral ischaemia.^6,7,10–15^ However, caveolae also have an important role in sensing changes in shear stress associated with cessation of blood flow, and subsequently regulating nitric oxide synthase activity and reactive oxygen species production in the lung.^51,52^ Therefore, the formation of caveolae after stroke may similarly be due to a reduction in blood flow, resulting not only in BBB breakdown, but initiation of the ischaemic cascade in endothelial cells.

## Supplementary Material

Supplementary material
